# Robotic Assisted Radical Cystoprostatectomy and Intracorporeal Ileal Conduit Urinary Diversion for a Kidney Transplant Recipient

**DOI:** 10.1590/S1677-5538.IBJU.2016.0227

**Published:** 2017

**Authors:** Peter A. Caputo, Daniel Ramirez, Matthew Maurice, Ryan Nelson, Onder Kara, Ercan Malkoc, David Goldfarb, Jihad Kaouk

**Affiliations:** 1 Departament of Urology Cleveland Clinic, Cleveland, Ohio, United States

## Abstract

**Introduction and Objectives:**

Robotic assisted radical cystectomy (RARC) is an alternative to open radical cystectomy. As experience is gained with the RARC approach the technique is being applied to more complex surgical cases. We describe here our technique for RARC with intracorporeal ileal conduit urinary diversion for a renal transplant recipient.

**Materials and Methods:**

The patient is a 60-year old man with high-grade muscle invasive bladder cancer. He has a history of renal failure due to polycystic kidney disease and received a deceased donor renal transplant in 2008. His hospital course at time of transplant was complicated by low-level BK virus viremia. Interestingly his trans-urethral bladder tumor resection specimen at time of bladder cancer diagnosis stained positive for SV40. His native kidneys were anuric so bilateral laparoscopic nephrectomy was performed in a staged fashion 2 weeks prior to RARC. Our surgical technique utilizes 6 trocars, of note a 12-mm assistant trocar is placed 1 cm superior to the pubic symphysis, and this trocar is solely used to pass a laparoscopic stapler to facilitate the excision of the ileal segment and the stapled enteric anastomosis. Surgical steps include: identification of native ureters bilaterally (removed *en bloc* with the bladder specimen); identification of the transplanted ureter at the right bladder dome; posterior bladder and prostate dissection along Denonvilliers’ fascia; development of the space of Retzius; ligation and transection of the bladder and prostate vascular bundles; apical prostate dissection and transection of urethra; left pelvic lymphadenectomy; ilium resection for creation of the ileal conduit; stapled enteric anastomosis; ureteroileal anastomosis; maturation of the ileal conduit stoma.

**Results:**

The surgery had no intraoperative complications. Operative time was 443 minutes (7.4 hours). Estimated blood loss was 250 cc. Length of hospital stay was 5 days. The patient did not experience any postoperative complications. The patient maintained good renal graft function with no decline in eGFR to date.

**Conclusions:**

As surgeon comfort and experience with robotic assisted surgery grows, robotic surgery can successfully be applied to less frequently performed procedures. Here we successfully performed a robotic assisted radical cystoprostatectomy with intracorporeal ileal conduit urinary diversion for a renal transplant recipient.

## ARTICLE INFO

**Available at: http://www.intbrazjurol.com.br/video-section/20160227-caputo_et_al**

**Int Braz J Urol. 2017; 43 (Video #16): 1192-1192**

